# Modeling the therapeutic dynamics of acupuncture and moxibustion: a systems biology approach to treatment optimization

**DOI:** 10.1016/j.csbj.2025.05.053

**Published:** 2025-06-01

**Authors:** Quan Gan, Qi-Wei Ge, Chuanxia Liu, Zhaoman Zhong, Jiaying Wu, Lei Shi, Jin Xu, Chen Li

**Affiliations:** a School of Computer Engineering, Jiangsu Ocean University, Lianyungang, China; b The Graduate School of East Asian Studies, Yamaguchi University, Yamaguchi-shi, Japan; c School of Foreign Languages, Jiangsu Ocean University, Lianyungang, China; d Department of Human Genetics, and Women’s Hospital, Zhejiang University School of Medicine & Zhejiang Provincial Key Laboratory of Genetic and Developmental Disorders, Hangzhou 310006, China

**Keywords:** Acupuncture and moxibustion treatment, Petri nets, Modeling, Simulation, Evaluation method

## Abstract

A key obstacle in advancing acupuncture and moxibustion treatment (AMT) lies in the absence of effective methodologies capable of modeling the body’s dynamic physiological changes and predicting treatment outcomes with quantitative precision. Colored Petri nets (CPNs), which have shown significant utility in simulating complex biological systems, offer a promising foundation for modeling AMT due to their capacity to represent hierarchical structures and dynamic behaviors. However, current modeling approaches struggle to address the inherent concurrency and complexity characteristic of AMT processes. To address this, we propose a novel token-guided transition control based on CPNs theory, enabling precise and efficient simulation of AMT systems. Furthermore, we develop a multicriteria evaluation method to quantitatively assess and compare the therapeutic efficacy of various AMT protocols, providing a structured approach for evidence-based decision-making. We validate our proposed model through simulation studies based on clinical cases of Meniere’s disease. The simulation results closely align with actual clinical data, supporting the model’s reliability and applicability. Finally, randomized simulation experiments have led to the identification of three new AMT strategies with promising therapeutic potential, highlighting the model’s capacity to support treatment optimization and clinical innovation. This study introduces a comprehensive framework for dynamic modeling, visual representation, and quantitative evaluation of AMT systems. By offering a systematic and predictive approach to AMT analysis, the proposed method not only enhances understanding of treatment mechanisms but also contributes to the standardization of clinical practice.

## Graphical Abstract

**Figure:**
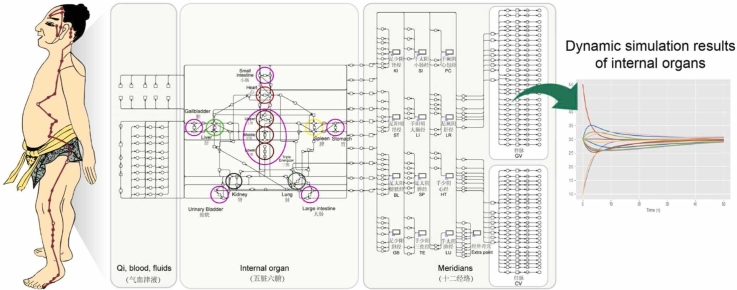

## 1 Introduction

Acupuncture and moxibustion treatment (AMT) has a long-standing history in clinical practice and is widely utilized for the management of complex chronic disorders, including pain syndromes, musculoskeletal conditions, and mental health disturbances. By delivering precise stimulation to designated acupoints, AMT is believed to regulate physiological pathways, promoting the restoration of Qi–blood balance and enhancing the body’s intrinsic healing and self-regulatory mechanisms [1], [2], [3], [4], [5]. The recognition of AMT in the World Health Organization (WHO)’s International Classification of Diseases in 2018 further highlights its clinical value and supports its integration into contemporary global healthcare systems. Nevertheless, despite its maturity in clinical application, AMT continues to face obstacles in achieving scientific standardization-particularly with respect to the quantification of organ-level interactions and the measurable impact of acupoint stimulation. These gaps hinder its assimilation into evidence-based medical paradigms and limit broader clinical endorsement [6], [7], [8], [9].

Since the mid-20th century, innovations in information science and digital technology have driven progress across numerous disciplines, including Traditional Chinese Medicine (TCM) [10], [11]. The emergence of computational modeling and system simulation has provided powerful tools to investigate internal physiological dynamics and systemic changes within the human body [12], [13]. Recently, research has increasingly focused on integrating technologies such as digital twins and computer simulation platforms with traditional TCM models. This integration has enabled advanced applications, including the three-dimensional visualization of meridians and acupoints [14], [15], high-resolution imaging of nerve and vascular structures surrounding acupoints [16], and interactive human-machine interfaces [17], [18], [19] that support risk-free training and simulation of AMT procedures. Additionally, methods such as 3D modeling, thermal distribution analysis, and investigations of bioelectrical signal transmission near acupoints [20], [21], [22] now facilitate the quantitative assessment of needling effects, offering novel insights into the physiological mechanisms underpinning AMT and expanding its potential for clinical innovation. However, these technological advancements have primarily focused on localized or isolated components of the treatment process. Current models often lack an integrated, system-level framework that reflects the complex, concurrent, and sequential interactions among organs, meridians, and acupoints. Furthermore, existing approaches do not provide robust quantitative methods for characterizing or analyzing these dynamic relationships holistically.

To address these limitations, this study introduces a novel modeling methodology designed to establish a quantitative characterization system that captures both organ interrelationships and the therapeutic effects of acupoint stimulation using Petri nets (PNs) [23], [24]. By translating traditional theoretical constructs into a computable and scientifically structured format, this approach creates an objective foundation for clinical decision-making, enabling precise prediction of therapeutic outcomes and supporting the standardization of AMT protocols. These findings may help bridge the gap between traditional therapeutic frameworks and modern evidence-based medicine, ultimately enhancing the clinical credibility, reproducibility, and global applicability of AMT.

## 2 Materials and methods

### 2.1 Modeling AMT system with colored Petri nets (CPNs)

#### 2.1.1 Brief introduction of CPNs

**Fig. 1:**
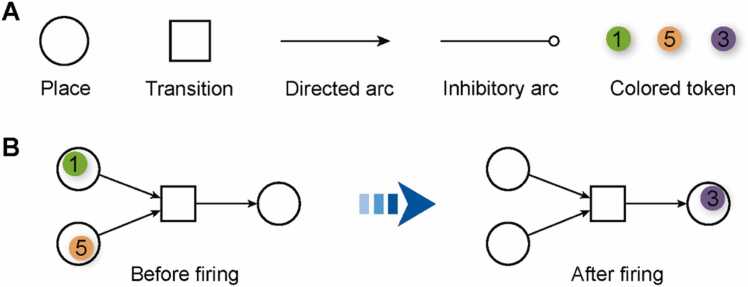
Basic elements and a simple example of the firing rules in CPNs.

CPNs are an advanced extension of classical PNs that incorporate a “color” attribute into *tokens*, enabling each token to carry specific data relevant to the modeled system. This feature allows for a richer and more detailed representation of system states and behaviors. Structurally, CPNs comprises two primary types of nodes—*places* and *transitions*—which are interconnected by either directed arcs or inhibitory arcs. Places function as containers for tokens, and the arrangement of these tokens across the network—referred to as a *marking*—captures the system’s current state. The directed arcs, often with associated weights, define both the preconditions required for transitions to fire and the postconditions resulting from those transitions, facilitating the representation of complex dynamic processes [23], [25]. CPNs are particularly well-suited for modeling intricate system behaviors, such as concurrency and sequentially. Concurrency refers to the system’s capacity to execute multiple events or actions simultaneously without interference, whereas sequentially ensures that specific actions occur in a predetermined order, with each subsequent event triggered only upon the successful completion of its predecessor [26]. These features make CPNs highly applicable across various domains, including system performance optimization, resource scheduling [27], [28], and systems biology [25], [29]. Formally, CPNs are defined by a 9-tuple notation: *CPNs* = ($\Sigma ,Ρ,Τ,F,V,C,G,E,M_{0}$) which corresponds to a bipartite graph representation. In this formulation, Σ denotes a set of color types (*REAL*, *INT*, *STRING, et al.*); Ρ is the set of places, typically illustrated as circles; Τ is the set of transitions, shown as bars or rectangles; F defines the set of directed arcs linking places to transitions and vice versa; *V* represents variables associated with arc expressions; *C* is the color function that assigns specific data types to places; *G* is the guard function, which assigns logical conditions to each transition t∈T, that must evaluate to “*True*” for the transition to occur; *E* represents the arc expression, specifying how tokens move through arcs; *M* denotes the marking, which maps each place *p* to a multiset of tokens and *M*_*0*_ represents the initial marking. Fig. 1 depicts the basic components and an example of the firing rules in CPNs.

We herein used CPN Tools, a specialized software platform developed for the construction, simulation, and analysis of CPNs models [30]. CPN Tools offers a user-friendly interface for building and refining model structures, supporting detailed parameter adjustments and dynamic simulation execution. The platform also provides comprehensive output logs and simulation datasets, enabling in-depth investigation of system behavior and performance trends. These features make CPN Tools a valuable resource for iterative model refinement and data-driven evaluation [31].

#### 2.1.2 AMT system

In TCM, disease is viewed as a consequence of internal organ imbalances, which AMT aims to correct by stimulating specific acupoints on the body. This stimulation—achieved through acupuncture needles or the application of heat via moxibustion—elicits physiological responses that promote the circulation of Qi and Blood through the meridian system, thereby targeting disorders associated with internal organ dysfunction. In TCM theory, the internal organs are classified into *five viscera* (the liver, heart, spleen, lungs, and kidneys) and *six bowels* (the gallbladder, small intestine, stomach, large intestine, urinary bladder, and triple energizer). Each viscus is associated with one of the five natural elements—wood, fire, earth, metal, or water—according to the Five Elements theory. The viscera and bowels are paired in functional relationships, such as “liver ↔ gallbladder” and “heart ↔ small intestine.”

The meridian system serves as the structural and functional bridge between the internal organs and external acupoints. These meridians are channels through which Qi flows, and they connect organ systems with specific points on the body surface [2], [6]. TCM identifies twelve primary meridians, each corresponding to a particular organ, as well as eight extra meridians that regulate and supplement the main channels. Each primary meridian is assigned a Yin or Yang designation and plays a specific physiological role—for example, the lung meridian (Hand Taiyin) is closely associated with respiratory function. To promote international standardization of acupuncture practices, the WHO introduced the “Standard Acupuncture Nomenclature,” which assigns unique abbreviations to each meridian based on its corresponding organ and limb location. For instance, “LU” denotes the lung meridian, while “LI” refers to the large intestine meridian [32]. Acupoints along each meridian are numbered sequentially to ensure consistent clinical documentation and research precision. For example, LU-1 indicates the first point on the lung meridian, whereas LI-4 identifies the fourth point on the large intestine meridian. Table 1 lists the names of each meridian with their WHO abbreviations, acupoint notations, and the total number of recognized points. The twelve primary meridians, along with the conception vessel and governor vessel meridians, contain 361 standard acupoints. In addition, 48 extra points located outside the meridian system are also acknowledged. These acupoints exert both local and systemic therapeutic effects by modulating the flow of Qi and enhancing physiological functions throughout the body [33].

**Table 1:** Meridians and Acupoints Notations.

Meridian name	WHO notation of meridian	WHO notation of acupoints	Number of acupoints
Lung Meridian of Hand-Taiyin	LU	LU1-LU11	11
Large Intestine Meridian of Hand-Yangming	LI	LI1-LI20	20
Stomach Meridian of Foot-Yangming	ST	ST1-ST45	45
Spleen Meridian of Foot-Taiying	SP	SP1-SP21	21
Heart Meridian of Hand-Shaoyin	HT	HT1-HT9	9
Small Intestine Meridian of Hand-Taiyang	SI	SI1-SI19	19
Bladder Meridian of Foot-Taiyang	BL	BL1-BL67	67
Kidney Meridian of Foot-Shaoyin	KI	KI1-KI27	27
Pericardium Meridian of Hand-Jueyin	PC	PC1-PC9	9
Triple Energizer Meridian of Hand-Shaoyang	TE	TE1-TE23	23
Gallbladder Meridian of Foot-Shaoyang	GB	GB1-GB44	44
Liver Meridian of Foot-Jueyin	LR	LR1-LR14	14
Conception Vessel Meridian	CV	CV1-CV24	24
Governor Vessel Meridian	GV	GV1-GV28	28
-	EX	EX-HN1-EX-HN15 EX-CA1 EX-B1-EX-B9 EX-UE1-EX-UE11 EX-LE1-EX-LE12	48

During the AMT process, the system demonstrates both concurrent and sequential behaviors. Concurrency is primarily reflected in the independent interactions between different viscera–bowel pairs (e.g., heart–small intestine and liver–gallbladder), wherein these relationships influence one another without mutual interference. Sequentially, by contrast, emerges from ordered physiological processes governed by TCM principles such as *mutual generation and restriction*, *exterior–interior organ relationships*, and *acupoint stimulation patterns*. According to TCM theory, the most fundamental relationship among the five viscera is the mutual generation and restriction cycle. In the exterior–interior framework, the viscera initiate functional commands to the bowels and receive regulatory feedback, producing a sequential relationship of viscera → bowels → viscera. Acupoint stimulation—an external therapeutic intervention—also follows this hierarchical structure and aligns with the exterior–interior organ paradigm.

In essence, the AMT system integrates static components (e.g., viscera, bowels, and acupoints) with dynamic processes (e.g., generation, restriction, and feedback), forming a system that simultaneously exhibits well-defined sequential flows (e.g., viscera → bowels → viscera) and concurrent interactions (e.g., among multiple viscera–bowel pairs). These dual characteristics render PNs particularly suitable for designing human physiological models and simulating AMT processes. The TCM conceptualization of the human body as a system governed by both concurrency and sequence closely aligns with the representational strengths of PNs modeling, which is well-equipped to capture the dynamic, cyclical nature of organ function and interconnectivity inherent to AMT [1], [2], [6].

#### 2.1.3 Construction of CPNs-based AMT simulation model

This study introduces a novel methodology for modeling the AMT system by integrating the Five Elements theory with fuzzy system theory to quantitatively represent internal organ states. Drawing from the findings in [34], the model assigns a numeric value within the range (0.5, 5.5) to each organ’s state, where [2.5, 3.5] denotes a balanced, healthy condition. Values in the range (0.5, 1.5) reflect weakness, while [1.5, 2.5) indicates mild weakness—both corresponding to deficiency. In contrast, (3.5, 4.5] signifies mild strength and (4.5, 5.5) reflects strength—both indicative of excess conditions. On this basis, eight equations were formulated to characterize dynamic changes during the AMT process.

(1) $$f_{H}\left(\tau^{\prime }\right)=g∙f_{L}\left(\tau -1\right)-r∙f_{K}\left(\tau -1\right)+s∙f_{H}\left(\tau -1\right)$$

(2) $$f_{\mathrm{SI}}\left(\tau^{\prime }\right)=c∙f_{H}\left(\tau^{\prime }\right)+s∙f_{\mathrm{SI}}\left(\tau -1\right)$$

(3) $$f_{H}\left(\tau^{\prime \prime }\right)=c∙f_{\mathrm{SI}}\left(\tau^{\prime }\right)+s∙f_{H}\left(\tau^{\prime }\right)$$

(4) $$f_{p}\left(\tilde{\tau }\right)=\mu \left(\tilde{\tau }\right)∙\left(e∙\frac{v\left(\tilde{\tau }\right)}{\left|v\left(\tilde{\tau }\right)\right|}+e∙v\left(\tilde{\tau }\right)\right)$$

(5) $$f_{V}\left(\hat{\tau }\right)=f_{V}\left(\tilde{\tau }\right)+w∙f_{p}\left(\tilde{\tau }\right)$$

(6) $$f_{i}\left(\hat{\tau }\right)=f_{i_{\mathrm{normal}}}∙\frac{\left(n∙h\right)-\left|\sum_{j\in O}f_{j}\left(\hat{\tau }\right)-\left(n\cdot h\right)\right|}{n∙h}\cdot \frac{h-\left|f_{\mathrm{me}}\left(\hat{\tau }\right)-h\right|}{h}$$

(7) $$f_{i\_V}\left(\hat{\tau }\right)=\frac{f_{i}\left(\hat{\tau }\right)}{v}∙\frac{\left(n∙h\right)-|\sum_{j\in O}f_{j}\left(\hat{\tau }\right)-\left(n\cdot h\right)|}{n∙h}∙\frac{h-|f_{\mathrm{ue}}\left(\hat{\tau }\right)-h|}{h}$$

(8) $$f_{V}\left(\tau \right)=f_{V}\left(\hat{\tau }\right)∙m+\left[f_{q\_V}\left(\hat{\tau }\right)∙q+f_{b\_V}\left(\hat{\tau }\right)∙b+f_{l\_V}\left(\hat{\tau }\right)∙l\right]$$

Eq. (1) captures the generating and restricting interactions among the five viscera. For example, the liver is influenced by the generative input from the heart, the restrictive influence from the kidney, and the self-degenerative effect of the heart itself. Eq. (2) models the exterior-to-interior regulation from viscera to bowels, while Eq. (3) reflects the reciprocal interior-to-exterior influence from bowels back to viscera. Eq. (4) incorporates different levels of acupuncture stimulation as external input, and Eq. (5) quantifies the direct effect of acupoint stimulation on internal organ states. Additionally, Eq. (6) simulates the generation of Qi, blood, and body fluids; Eq. (7) models the transport of these vital substances throughout the body, and Eq. (8) describes their absorption by internal organs, contributing to the maintenance of physiological balance. In these equations, *f* denotes the organ state values, while parameters such as *g*, *r*, *s*, *c*, *e*, *w*, *h*, *v*, *m*, *q*, *b,* and *l* represent the intensity of various influences exerted by internal organs, acupoints, and Qi–blood–body fluid dynamics. Based on the conclusions of [34] and extensive trial-and-error calibrations, these parameters are assigned as follows: *g =* 0.15, *r* = 0.05, *s* = 0.9*, c =* 0.1*, e =* 0.25*, w =* 0.01*, h =* 3*, v =* 10*, m =* 0.999*, q =* 0.004*, b =* 0.004*, l =* 0.002.

To simulate temporal changes in organ states, a time parameter, *τ*, is introduced. Each simulation cycle (τ−1 to τ), involves the sequential computation of (1), (2), (3), (4), (5), (6), (7), (8) in the following order: Mutual generation and restriction among the five viscera → Influence of viscera on bowels → Feedback from bowels to viscera → Acupuncture-induced stimulation of internal organs → Effects of Qi–blood–body fluid generation, transport, and absorption. To maintain consistency in process progression, distinct temporal notations such as ($\tau^{\prime },\tau^{\prime \prime },\tilde{\tau },\hat{\tau }$) are used to differentiate stages, with the hierarchy τ>τ−1, ensuring proper chronological flow in simulation.

**Fig. 2:**
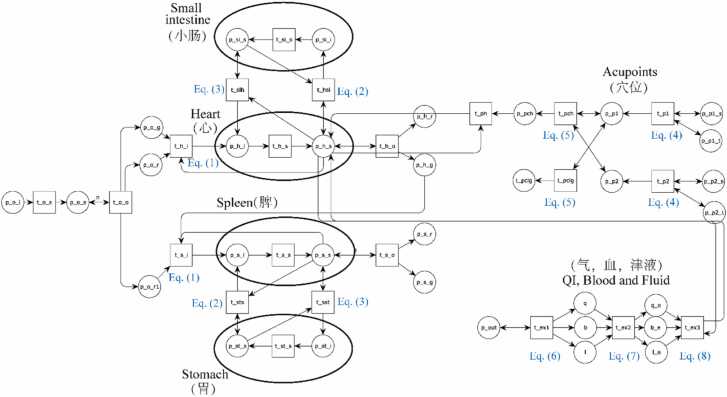
CPNs models of the AMT system (partial).

The AMT simulation model was constructed using CPN Tools, allowing integration of internal organ dynamics, acupoint stimulation, and Qi–blood–body fluid regulation [35], [36], [37]. A partial representation of the model is depicted in Fig. 2. In this model, the places $p_{\mathrm{si\_s}}$ and $p_{\mathrm{si\_i}}$, along with the transition $t_{\mathrm{si\_s}}$, represent a specific organ (e.g., the small intestine). The transitions $t_{\mathrm{sih}}$ and $t_{\mathrm{hsi}}$ link viscera–bowel pairs and embed (2), (3) to simulate their bidirectional interactions. The transition $t_{\mathrm{h\_i}}$ connects three viscera and implements Eq. (1), modeling the generative and restrictive relationships defined by the Five Elements theory.

#### 2.1.4 Token-guided transition control (TGTC) method for solving CPNs concurrency problems in AMT system

Despite the incorporation of the time parameter τ in (1), (2), (3), (4), (5), (6), (7), (8), implementing a strictly time-ordered computation within the CPNs framework remains a significant modeling challenge. To overcome this, we introduce a novel mechanism termed the TGTC method. This approach embeds auxiliary control structures directly into the simulation architecture to inherently manage the order and concurrency of transitions, ensuring that the model accurately reflects the temporal and logical sequence of processes in the AMT system. The TGTC method introduces three core auxiliary elements—*control places*, *control tokens*, and *control arcs*—which collectively regulate the execution logic of transitions. These elements function as follows:

(1) Control place addition: A unique control place is assigned to each transition. These control places hold specially designated control tokens, which act as conditional triggers. A transition cannot fire unless the corresponding control token is present in its control place, thereby embedding execution constraints into the model's structure.
(2) Sequential control: To simulate time-sequential processes, a control token is first placed in the control place of the initial transition. A control arc connects this control place to the transition, allowing it to fire. Upon execution, the control token is transferred to the control place of the subsequent transition in the sequence. This chain of controlled handoffs ensures that transitions are fired in a strictly defined order, reflecting the stepwise nature of physiological processes modeled in AMT.
(3) Concurrent control: In cases where transitions represent independent or parallel physiological processes—such as interactions among different viscera–bowel pairs—concurrent control is applied. A control token is placed in the starting transition’s control place, and when this transition fires, the token is split via multiple control arcs into different execution paths. Each path represents a distinct process that runs in parallel. At the termination point of each concurrent branch, a control place is introduced to collect the control tokens. The output arcs of these endpoint transitions converge at a common control place, which signifies the completion of all concurrent subprocesses and permits subsequent sequential actions to proceed.

**Fig. 3:**
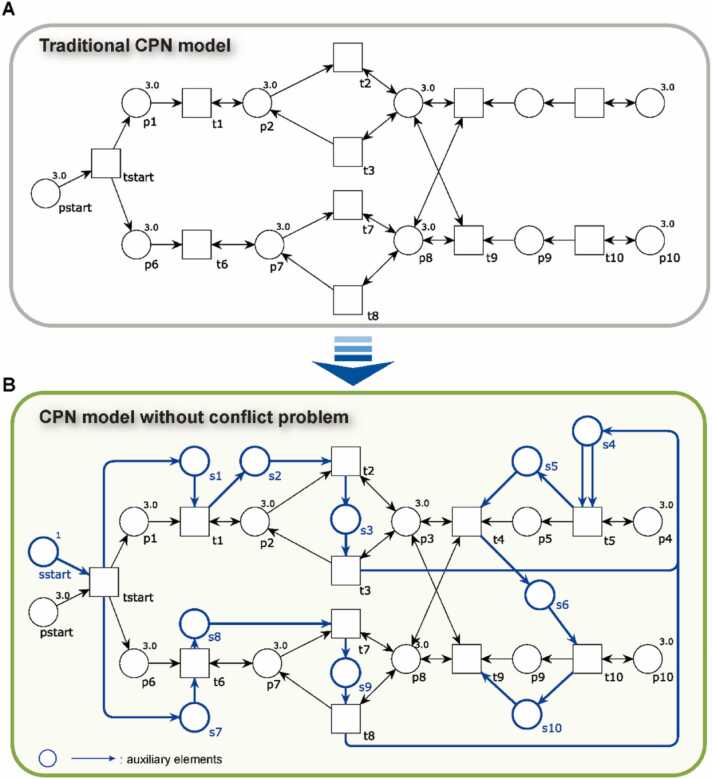
An example illustrating a new modeling approach of solving CPNs concurrency problems in the AMT system.

As illustrated in Fig. 3A, transitions capable of firing simultaneously (i.e., the equations that can be executed) include $t_{1}$-$t_{3}$,$\;t_{5}$-$t_{8}$ and $t_{10}$. However, this unrestricted concurrency fails to reflect the intended physiological sequence inherent to the AMT process. To address this discrepancy, Fig. 3B demonstrates how the introduction of auxiliary control elements restructures the firing sequence to more accurately mirror the physiological progression observed during AMT. In this refined model, the transition *t*_*start*_ initiates the process. Upon firing, it activates transitions $t_{1}$ and $t_{6}$, both of which represent Eq. (1) (mutual generation and restriction among the five viscera), provided their input conditions—namely, the presence of requisite tokens—are satisfied. These two transitions initiate two concurrent subprocesses: $t_{1}$→$t_{2}$→$t_{3}$ and $t_{6}$→$t_{7}$→$t_{8}$. These paths simulate parallel organ interactions within the Five Elements framework, illustrating simultaneous generative and restrictive relationships between internal organs. Upon the completion of these concurrent sequences, the model transitions into a downstream phase—$t_{5}$→$t_{4}$→$t_{10}$→$t_{9}$—which captures the effects of acupoint stimulation and Qi-blood-body fluid dynamics. This sequence represents the body's systemic response to AMT, incorporating both neural and humoral regulatory mechanisms. Notably, the model defines two types of places—depicted in black and blue—that carry distinct attributes. Although these places may be triggered by the same transition (e.g., *t*_*start*_), their state variables are functionally independent. For instance, a token in *sstart* does not influence the behavior or availability of a token in *pstart*, maintaining separation between structural and regulatory pathways.

Through the TGTC framework, the model achieves a synchronized blend of sequential control and concurrent execution. By embedding auxiliary control logic directly into the CPNs' structure, the simulation accurately reflects the hierarchical and temporal dependencies of physiological processes, thereby offering a realistic and interpretable model of AMT function.

### 2.2 Modeling and simulation of AMT for Meniere's disease: clinical case study

#### 2.2.1 Clinical background of AMT for Meniere's disease

Meniere’s disease is an inner ear disorder marked by vertigo, tinnitus, and hearing loss, with its exact cause still unclear. AMT has shown promise in alleviating symptoms by stimulating specific acupoints [38]. Clinical application of AMT in Meniere’s disease identifies the underlying pattern as liver excess with spleen and kidney deficiency. The treatment protocol involves stimulation of *GV20* (*Baihui*, *Governor Vessel*), GV16 (*Fengfu*, *Governor Vessel*), *GB20* (*Fengchi*, *Gallbladder Meridian*), *BL23* (*Shenshu*, *Bladder Meridian*), *KI3* (*Taixi*, *Kidney Meridian*), and *GB39* (*Xuanzhong*, *Gallbladder Meridian*). Among these, GB20 is treated with purgation, while the others are treated with tonification. All acupoints are stimulated at normal intensity [39].

#### 2.2.2 Exploration of AMT for Meniere’s disease

To more comprehensively evaluate the simulation model’s utility, we systematically explored AMT protocols for Meniere’s disease that have not yet been documented. Based on three distinct selection approaches, we simulated various combinations of acupoints and stimulation levels to identify effective treatment alternatives beyond those currently documented. The three selection approaches are as follows:

• Completely Random (Group R): Acupoints and stimulation levels were randomly selected without overlap with known treatments.
• Meridian-Based Selection (Group M): Acupoints were selected according to TCM meridian theory, targeting meridians related to the affected organ (e.g., selecting liver meridian points for liver-related issues).
• Correspondence-Based Selection (Group C): Acupoints were chosen based on TCM organ pair relationships (e.g., selecting gallbladder meridian points for liver-related issues).

Five rounds of random selection were performed for each group, labeled R1–R5, M1–M5, and C1–C5. Acupoint sets and stimulation levels are detailed in Table 2. For instance:

• R1: The selected acupoints were KI8: −5, SP1: −3, HT9: −1, SI18: 4, BL62: 2, GB19: 1, and CV2: −2.
• M1: The selected acupoints included LR1: −3, LR14: 3, SP2: 3, SP19: −3, KI10: −3, and KI21: 3 selected from the liver, spleen, and kidney meridians.
• C1: The selected acupoints included GB20: −3, GB25: 3, BL40: 3, BL55: −3, ST40: −3, and ST45: 3 selected from gallbladder, stomach, and bladder meridians.

**Table 2:** Acupoint and stimulation levels for each group.

Group	Acupoint: stimulation levels
R1	KI8: −5, SP1: −3, HT9: −1, SI18: 4, BL62: 2, GB19: 1, CV2: −2
R2	KI16: −5, LU8: 2, ST10: 1, HT2: 5, BL7: −1, SP3: −5
R3	GB14: 4, PC7: 3, TE22: −4, GV28: −2, CV6: −1
R4	CV24: 5, BL49: −5, SI12: 3, SP16: −3, ST41: 1, LI20: −1
R5	EXHN4: 4, EXB3: −4, LU1: −2, LR2: 2, KI9: −3
M1	LR1: −3, LR14: 3, SP2: 3, SP19: −3, KI10: −3, KI21: 3
M2	LR6: 3, LR12: −3, SP6: −3, SP21: 3, KI1: 3, KI3: −3
M3	LR10: −3, LR11: 3, SP10: 3, SP16: −3, KI15: −3, KI19: 3
M4	LR12: 3, LR3: −5, SP17: 4, SP18: −2, KI23: 3, KI20: −4
M5	LR2: 4, LR14: −4, SP4: −1, SP5: 2, KI17: −4, KI27: 1
C1	GB20: −3, GB25: 3, BL40: 3, BL55: −3, ST40: −3, ST45: 3
C2	GB1: 3, GB10: −3, BL15: −3, BL25: 3, ST38: 3, ST39: −3
C3	GB40: 3, GB44: −3, BL60: −3, BL65: 3, ST1: 3, ST10: −3
C4	GB1: 4, GB26: −5, BL36: 3, BL21: −2, ST2: 2, ST10: −1
C5	GB43: 5, GB26: −4, BL20: −5, BL41: 3, ST6: 4, ST42: −4

This exploration highlights the model’s ability to replicate physiological dynamics, offering a platform to test and expand AMT strategies for Meniere’s disease. Through testing diverse combinations of acupoints, the study aims to broaden potential treatment options and validate the effectiveness of simulation-guided approaches.

#### 2.2.3 Simulation procedures

In AMT simulations, parameter settings were based on clinical principles for treating Meniere’s disease. According to these principles, the liver is typically in a state of excess, whereas the spleen and kidneys are considered deficient. Accordingly, the simulation model assigned an initial value of 5 to the liver and 1 to both the spleen and kidney. Other internal organs, assumed to be in a healthy or balanced state, were each set to an initial value of 3.

Acupoint stimulation was configured as follows: GB20 was treated with normal-intensity purgation, represented by a value of −3. All other stimulated acupoints were assigned a value of 3, reflecting normal-intensity tonification. Acupoints not involved in treatment were given a neutral value of 0.

Once these parameters were input into the simulation model, the number of iterations was set to 50 (*τ* = 0 to *τ* = 50), corresponding to the time steps of a single case. Each simulation was completed in approximately 2 seconds and recorded the internal organ states at each time step. Simulations were run on a system equipped with an Intel(R) Core(TM) i7–13700KF processor (3.40 GHz) and 32.0 GB of RAM.

#### 2.2.4 Performance evaluation

To identify promising treatments from randomly generated acupoint combinations, a rigorous evaluation method was applied. This method integrated three key clinical criteria: (1) the stability of internal organ health states, (2) whether all organs remained within a healthy range, and (3) the degree of similarity to established treatment profiles. Stability was evaluated by calculating the proportion of time each organ’s state remained within the healthy range throughout the simulation. For the second criterion, the minimum root mean square deviation (RMSD) of each health state was calculated and compared to a predefined threshold to confirm all values stayed within acceptable limits. The third criterion measured similarity to existing treatments by defining an envelope of maximum and minimum values for each time point, based on reference simulations, and computing the proportion of time the test simulation results remained within this envelope, adjusted for an acceptable margin of error. This multicriteria framework ensures that newly generated acupoint combinations are thoroughly assessed for clinical relevance. The overall evaluation score is computed using the following formula:

(9) $$E\mathrm{val}\left(P_{\mathrm{TR}},f_{\mathrm{RMSD}},P_{\mathrm{ET}}\right)=P_{\mathrm{TR}}∙m+f_{\mathrm{RMSD}}∙n+P_{\mathrm{ET}}∙k$$

where $P_{\mathrm{TR}}$ indicates whether the internal organ states remained stable, $f_{\mathrm{RMSD}}$ represents whether all states stayed within healthy limits, and *P_ET* measures similarity to reference treatments. The calculation procedures for $P_{\mathrm{TR}}$, $f_{\mathrm{RMSD}}$, and $P_{\mathrm{ET}}$ are detailed in Eqs. (10–12) and further explained in Supplementary Information (Appendix Information A). The corresponding weights are *m =* 0.4, *n* = 0.4*,* and *k* = 0.2.

In summary, this evaluation method integrates three clinically meaningful indicators and produces a composite score via Eval(). A higher score suggests a greater likelihood of treatment efficacy, while a lower score indicates limited clinical potential. This framework was applied to the simulation groups described in Section 2.2.2 to identify novel treatment candidates.

## 3 Results

### 3.1 Modeling AMT system for Meniere’s disease

**Fig. 4:**
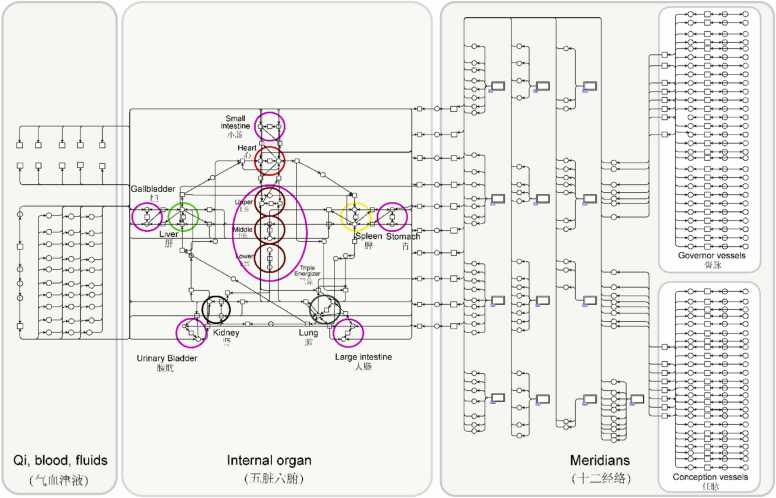
The entire CPNs-based AMT system model for Meniere’s disease.

The complete CPNs-based AMT system model is shown in Fig. 4. It encompasses all human body components involved in the AMT system, including internal organs, Qi-blood-body fluids, meridians, and acupoints. The model comprises a total of 2459 places, 713 transitions, and 3948 arcs. To manage the scale and complexity of the model effectively, the hierarchical functionality of CPN Tools is employed to modularize and organize its elements. This feature enables certain CPNs elements to be grouped into compact units—such as the double-bordered rectangle representing a single meridian in Fig. 4—without altering the model’s structure or internal connections. The model is organized into multiple layers. The first layer depicts the overall AMT system, which is subdivided into two modules in the second layer: the internal organs module and the meridian module. The internal organs module contains the Qi, blood, and body fluids module at the third layer. Likewise, the meridian module includes 14 individual meridian modules and an extra points module, also located at the third layer.

### 3.2 Effectiveness of simulation model and evaluation method

**Fig. 5:**
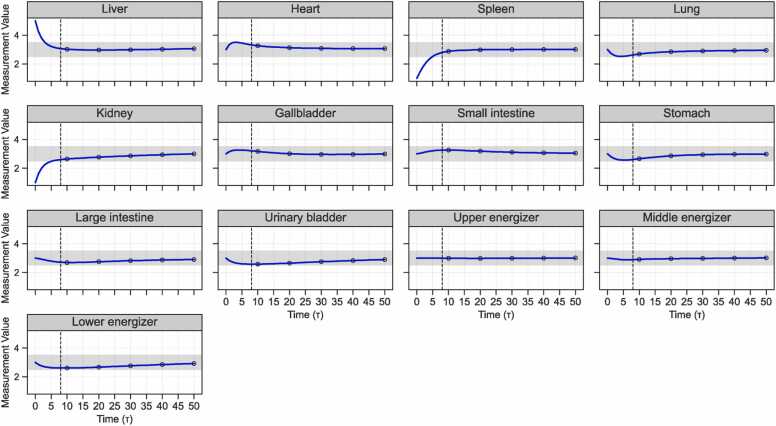
Dynamic changes in the internal organs during AMT for Meniere's disease.

Using the constructed simulation model, we conducted a case simulation of AMT for Meniere’s disease, with the results shown in Fig. 5. The X-axis represents the number of simulation iterations (*τ*), and the Y-axis denotes the state values of the internal organs, ranging from 0.5 to 5.5. As illustrated, the liver, spleen, and kidney states improve rapidly as *τ* increases. By *τ* = 8, all three organs reach the healthy range (2.5–3.5), after which their values stabilize around 3.0 with minimal fluctuation. This dynamic pattern aligns well with clinical observations, thereby supporting the model’s capacity to accurately simulate the therapeutic effects of AMT in practice.

The same simulation process was applied to the randomly generated acupoint combinations introduced in Section 2.2.2 The resulting data were assessed using the comprehensive evaluation method. Table 3 summarizes the evaluation results. The reference group (Group Reference), which followed a clinically validated treatment protocol, achieved an *Eval()* score of 0.960, confirming its effectiveness. Among the exploratory groups, R4, M1, and C1 attained relatively high scores of 0.804, 0.840, and 0.804, respectively, indicating promising therapeutic potential. In contrast, combinations such as R1, R2, and C5 scored below 0.1, suggesting limited or no expected clinical benefit. These findings reinforce the robustness and discriminative power of the evaluation framework, highlighting its value in identifying potentially effective acupoint combinations through simulation-guided strategies.

**Table 3:** Evaluation outcomes of simulation results from distinct acupoint combinations.

Group	$P_{\mathrm{TR}}$	$f_{\mathrm{RMSD}}$	$P_{\mathrm{ET}}$	*Eval*()
Ref [39]	0.90	1	1.00	0.960
R1	0.00	0	0.06	0.012
R2	0.00	0	0.14	0.028
R3	0.76	1	0.36	0.776
R4	0.82	1	0.38	**0.804**
R5	0.76	1	0.34	0.772
M1	0.74	1	0.72	**0.840**
M2	0.00	0	0.56	0.112
M3	0.40	1	0.24	0.608
M4	0.48	0	0.36	0.264
M5	0.00	1	0.04	0.408
C1	0.86	1	0.30	**0.804**
C2	0.84	1	0.06	0.748
C3	0.60	1	0.24	0.688
C4	0.78	0	0.46	0.404
C5	0.00	0	0.10	0.002

In summary, the simulation model developed in this study, when integrated with a clinically validated AMT treatment protocol, successfully replicates the dynamic physiological changes induced by AMT. The model demonstrates a high degree of fidelity in reflecting real-world clinical outcomes, underscoring its authenticity and reliability. Additionally, the accompanying evaluation method effectively differentiates the therapeutic impacts of various acupoint combinations by quantifying their influence on internal organ states. This enables a systematic and data-driven assessment of their potential clinical efficacy, offering valuable insights for optimizing acupoint selection in AMT practice.

## 4 Discussion

### 4.1 Methodological contributions and practical implications

This study employed CPNs, a powerful mathematical modeling framework, to capture the complex concurrent and sequential dynamics inherent in AMT. The proposed model was first validated using a clinically established AMT protocol for Meniere’s disease, with simulation results demonstrating strong agreement with real-world clinical outcomes. To evaluate the model’s generalizability, we further simulated a published clinical case of night terror [40]. The parameter configurations and corresponding simulation outcomes are detailed in Supplementary Information (Appendix Information B) to preserve the clarity and focus of the main text. The results of this additional case also closely aligned with clinical observations, further confirming the model’s applicability across a range of pathological conditions. In addition to the simulation model, we developed a novel evaluation function that quantitatively assesses the therapeutic efficacy of AMT interventions. This function provides a computationally rigorous method for evaluating treatment outcomes and enhances the model’s utility as a decision-support tool.

Our study introduces a new quantitative paradigm for understanding and assessing AMT, addressing the current absence of standardized clinical evaluation metrics. By representing organ–acupoint interactions within the CPNs framework, we enable a data-driven simulation and analysis of therapeutic mechanisms from an information science perspective. This approach facilitates the prediction of treatment efficacy, the comparative evaluation of alternative AMT strategies, and the identification of optimal acupoint combinations tailored to specific clinical scenarios. Moreover, by benchmarking against clinically validated protocols, our evaluation framework robustly assesses the feasibility and effectiveness of randomized acupoint combinations across multiple dimensions, offering valuable guidance for both clinical practitioners and researchers. Compared with previous work, this study advances the AMT modeling framework in two significant ways. First, we incorporate critical physiological components—namely Qi, blood, and body fluids—that were previously excluded, thereby constructing a more holistic representation of human physiological processes. Second, unlike earlier studies that applied auxiliary regulatory elements only in isolated instances, we generalize the control mechanism by introducing the TGTC method. This innovation yields a unified, reusable, and scalable control architecture capable of regulating the entire system, thus enhancing the model's extensibility and applicability across diverse therapeutic contexts.

### 4.2 Limitations and future work

Although the proposed simulation model has shown strong potential in replicating clinical outcomes of AMT, several limitations remain and warrant further development. First, the accuracy and robustness of the model could be significantly enhanced by incorporating a broader and more diverse range of clinical data. This would allow for the refinement of existing formula parameters, enabling more precise simulations that account for interindividual variability and the nuanced physiological responses observed in clinical practice. Second, the current model does not include the eight extraordinary meridians, which are considered fundamental in TCM for regulating and integrating the functions of the primary meridians. Incorporating these additional meridians into the PNs framework will be a key focus in future work, providing a more comprehensive and authentic representation of the AMT process. Third, to further evaluate the generalizability and clinical relevance of the model, future research will involve extensive simulation studies using a wider array of clinical cases across different disease types and syndromes. This expansion will help determine the model’s applicability across diverse pathological contexts and support its use in a broader clinical setting. In addition, we aim to develop a knowledge-based reasoning algorithm for acupoint selection. Building upon the current findings, this algorithm would automatically generate and evaluate alternative treatment schemes. Such a system could facilitate clinical decision-making by recommending personalized AMT plans tailored to the specific conditions and characteristics of individual patients. Finally, while the composite evaluation function proposed in this study offers a promising tool for assessing therapeutic efficacy, it currently lacks empirically derived cutoff values. To enhance its clinical interpretability and utility, future studies will integrate clinical outcome data and apply appropriate statistical methods to establish meaningful thresholds for evaluation scores.

In summary, the simulation model developed in this study provides a powerful and dynamic computational framework for advancing both the theoretical and clinical understanding of AMT. By quantitatively modeling the physiological effects of AMT, the model supports the evaluation of existing therapeutic protocols and facilitates the exploration of novel treatment strategies. Continued development—through the integration of clinical datasets, expansion of physiological scope, and refinement of evaluation mechanisms—will further strengthen its potential as a decision-support tool in both research and clinical practice.

## Author statement

We hereby declare that the manuscript entitled “Modeling the Therapeutic Dynamics of Acupuncture and Moxibustion: A Systems Biology Approach to Treatment Optimization” is original, has not been published previously, and is not under consideration for publication elsewhere. All named authors have read and approved the final version of the manuscript, and no other individuals meeting the criteria for authorship have been omitted. We designate the Corresponding Author as the sole contact for all editorial matters; she will coordinate communications with co-authors regarding submission progress, revision requests, and final proof approval.

## CRediT authorship contribution statement

**Chen Li:** Writing – review & editing, Writing – original draft, Supervision, Methodology, Conceptualization. **Jiaying Wu:** Visualization, Supervision. **Zhaoman Zhong:** Formal analysis, Data curation. **Jin Xu:** Investigation, Data curation. **Lei Shi:** Investigation, Conceptualization. **Quan Gan:** Writing – original draft, Visualization, Methodology, Conceptualization. **Chuanxia Liu:** Validation, Formal analysis. **Qi-Wei Ge:** Methodology, Conceptualization.

## Declaration of Competing Interest

We declare that we have no financial and personal relationships with other people or organizations that can inappropriately influence our work, there is no professional or other personal interest of any nature or kind in any product, service and/or company that could be construed as influencing the position presented in, or the review of, the manuscript entitled.

## Footnotes

Appendix A Supplementary data associated with this article can be found in the online version at doi:10.1016/j.csbj.2025.05.053.

## References

[1] Zheng H., Wang Z., Li S. (2020). Effect of acupoints on acupuncture-moxibustion and its therapeutic mechanism. World J Tradit Chin Med.

[2] Ren M., Liu Y., Ni X. (2022). The role of acupuncture and moxibustion in the treatment, prevention, and rehabilitation of patients with COVID-19: a scoping review. Integr Med Res.

[3] Chon T.Y., Lee M.C. (2013). Acupuncture. Mayo Clin Proc.

[4] Vanderploeg K., Yi X. (2009). Acupuncture in modern society. J Acupunct Meridian Stud.

[5] Sun M.-z, Wang X., Li Y.-c (2024). Effects of acupuncture needle modification on acupuncture analgesia. J Integr Med.

[6] Liu W., Chen C., Wang F. (2020). Development trend and current situation of acupuncture-moxibustion indications. World J Acupunct-Moxibustion.

[7] Guo Y., Wang J., Chen B. (2020). Comput Acupunct. World Chin Med.

[8] Liu B., Chen B., Guo Y. (2021). Acupuncture–a national heritage of China to the world: international clinical research advances from the past decade. Acupunct Herb Med.

[9] Wang Y., Shi X., Efferth T. (2022). Artificial intelligence-directed acupuncture: a review. Chin Med.

[10] Wu J., Koelzer V.H. (2024). Towards generative digital twins in biomedical research. Comput Struct Biotechnol J.

[11] Ren Z., Ren Y., Li Z. (2024). TCMM: A unified database for traditional Chinese medicine modernization and therapeutic innovations. Comput Struct Biotechnol J.

[12] Kardynska M., Kogut D., Pacholczyk M. (2023). Mathematical modeling of regulatory networks of intracellular processes - Aims and selected methods. Comput Struct Biotechnol J.

[13] Monteiro M., Fadda S., Kontoravdi C. (2023). Towards advanced bioprocess optimization: a multiscale modelling approach. Comput Struct Biotechnol J.

[14] Liu C., Zhang J., Wang J. (2021). Design and implementation of acupoint simulation system of mobile digital human body based on Unity3D technology. Mod Tradit Chin Med Mater Med - World Sci Technol.

[15] Gao H., Gao Y., Kan H. (2020). Research on development and design of virtual acupuncture teaching software based on HTC VIVE. Microcomput Appl.

[16] Hu W., Chen J., Sun C. (2021). Spatial topological analysis of sympathetic neurovascular characteristic of acupoints in Ren meridian using advanced tissue-clearing and near infrared II imaging. Comput Struct Biotechnol J.

[17] Xu Y., Zhang P., Wang Z. (2018). A Method of Interactive Virtual Human Body Meridian Acupoints System Simulation and Implementation Based on MFC and OpenGL. Jisuanji Yu Xiandaihua.

[18] Yu H., Zhu Z., Wang C. (2024). Kinematic-driven human-robot interaction system with deep learning for flexible acupuncture needling manipulations. Biomed Signal Process Control.

[19] Du G., Li Y., Su K. (2023). A mobile natural human–robot interaction method for virtual chinese acupuncture. IEEE Trans Instrum Meas.

[20] Liu Y., Yan Z., Guo Y. (2016). Virtual three-dimension reconstruction of Sìdú (te9). World J Acupunct-Moxibustion.

[21] Jin W., Tao Y., Wang C. (2023). Infrared imageries of human body activated by tea match the hypothesis of meridian system. Phenomics.

[22] Ma Q. (2022). Somatotopic organization of autonomic reflexes by acupuncture. Curr Opin Neurobiol.

[23] Peterson J. (1981). Petri Net Theory and the Modeling of Systems.

[24] Yu Y.-X., Gong H.-P., Liu H.-C. (2023). Knowledge representation and reasoning using fuzzy Petri nets: a literature review and bibliometric analysis. Artif Intell Rev.

[25] Assaf G., Heiner M., Liu F. (2022). Coloured fuzzy Petri nets for modelling and analysing membrane systems. Biosystems.

[26] Ohta A., Tsuji K. (2011). Net Theory. IEICE ESS Fundam Rev.

[27] Verma P., Sood S.K., Kaur H. (2024). Data driven stochastic game network-based smart home monitoring system using IoT-enabled edge computing environments. IEEE Trans Consum Electron.

[28] Santos L., Brito C., Fé I. (2024). Event-based moving target defense in cloud computing with VM migration: a performance modeling approach. IEEE Access.

[29] Liu F., Yamamoto E., Shirahama K. (2022). Analysis of pattern formation by colored petri nets with quantitative regulation of gene expression level. IEEE/ACM Trans Comput Biol Bioinform.

[30] Ratzer A.V., Wells L., Lassen H.M. (2003). International conference on application and theory of petri nets.

[31] Nickaeen N., Moein S., Heidary Z. (2016). Colored petri net modeling of small interfering RNA-mediated messenger RNA degradation. Adv Biomed Res.

[32] Lim S. (2010). WHO Standard Acupuncture Point Locations. Evid Based Complement Altern Med.

[33] Jung H., Lim S., Jung H. (2018). Study on the nomenclature of acupoints for the healthcare terminology standard. J Korean Med.

[34] Sun C., Li X., Zhao L. (2011). Fuzzy modeling and analysis based on five elements theory for the system of five organs system. J Anshan Norm Univ.

[35] Ge Q., Wu R., Nakata M. (2015). On Modeling internal organs and meridian system based on traditional Chinese medicine. BioPPN@ Petri Nets.

[36] Gan Q., Wu R., Nakata M. (2021). Construction of a human body model for acupuncture and moxibustion treatment by colored Petri nets. Biosystems.

[37] Gan Q., Wu R., Nakata M. (2021). Construction of human body model by colored petri nets based on traditional Chinese medicine. IEICE Trans Inf Syst.

[38] Gong Y., Yan Z., Feng W. (2024). Expert consensus on clinical diseases responding specifically to traditional Chinese medicine: aural vertigo. Chin J Exp Tradit Med Formula.

[39] Luan Y., Zhou X., Gong Z. (2008). Treatment of 32 cases of Meniere disease with acupuncture. J Acupunct Tuina Sci.

[40] Nakamura M., Takahashi R., Sakaguchi S. (2019). Effects of acupuncture therapy on 83 cases of infants with night terrors. J Jpn Soc Acupunct Moxibustion.

