# Supplementary material for: Modeling the therapeutic dynamics of acupuncture and moxibustion: a systems biology approach to treatment optimization
Source: Comput Struct Biotechnol J. 2025 Jun 1;27:2434–42. doi: 10.1016/j.csbj.2025.05.053 (PMC12174566; doi:10.1016/j.csbj.2025.05.053)
Supplement: Supplementary file 1 — Supplementary material [file mmc1.docx]

**Supplementary Material**

**Modeling the Therapeutic Dynamics of Acupuncture and Moxibustion: A Systems Biology Approach to Treatment Optimization**

Quan Gan, Qi-Wei Ge, Chuanxia Liu, Zhaoman Zhong, Jiaying Wu, Lei Shi, Jin Xu, Chen Li

# Appendix Information A

In Eq. (9), deriving the *Eval ()* score requires computing the three parameters $P_{TR}$, $f_{RMSD}$, and $P_{ET}$. For $P_{TR}$, the calculation method is defined in Eq. (10), where $Count(\tau)$ represents the duration during which all state values remain within the healthy range, and $\tau_{all}$ denotes the total simulation time. The set $O$ refers to the collection of internal organs, and the indicator function $\boldsymbol{1}\left( 2.5\leq f_{i}\left( \tau_{j} \right)\leq3.5,\forall i\in O \right)$ represents the condition is satisfied. The summation $\sum_{j=1}^{\tau_{all}}$is used to count the total duration.

$\left\{ \begin{aligned} P_{TR}=\frac{Count\left( \tau\right)}{\tau_{all}} \\ Count\left( \tau\right)=\sum_{j=1}^{\tau_{all}} \boldsymbol{1}\left( 2.5\leq f_{i}\left( \tau_{j} \right)\leq3.5,\forall i\in O \right) \end{aligned} \right.$ (10)

For $f_{RMSD}$, the calculation method is shown in Eq. (11), where $\sigma$ represents the RMSD, and $\sigma_{min}$ denotes the minimum value of $\sigma$ over the simulation time $\tau$. Additionally, $\sigma_{limit}$ indicates the limit value, specifically the $\sigma$ value when all viscera status values reach their boundary values (2.5 or 3.5). The indicator function evaluates to 1 when $\sigma_{min}\leq\sigma_{limit}$; otherwise, it evaluates to 0. The set $O$ represents the collection of internal organs, $f_{Health}$ denotes the optimal health value, and $Count(O)$ is the total number of internal organs.

$$\left\{ \begin{aligned} f_{RMSD}(\sigma_{min},\sigma_{limit})=\{\begin{matrix} 1, & If \sigma_{min}\leq\sigma_{limit} \\ 0, & If \sigma_{min}>\sigma_{limit} \end{matrix} \\ \sigma_{Min}=Min\left( \sigma_{1},\sigma_{2}\ldots\sigma_{j} \right) \\ \sigma_{j}=\sqrt{\frac{\sum_{i=1}^{i\in O} (f_{i}\left( \tau_{j} \right)-f_{Health})^{2}}{Count\left( O \right)}},\left\{ j\in\mathbb{Z}^{+} \mid1\leq j\leq\tau\right\} \end{aligned} \left( 11 \right) \right.$$

The calculation method for $P_{ET}$ is shown in Eq. (12), where $L_{E_{j}}$ and $U_{E_{j}}$ represent the upper and lower boundaries of the envelope, and $L_{R_{j}}$ and $U_{R_{j}}$ represent the upper and lower boundaries of the simulation results, respectively. The indicator function $\boldsymbol{1}(L_{E_{j}}\leq L_{R_{j}}\leq U_{R_{j}}\leq U_{E_{j}})$ represents the envelopment test condition is satisfied. The summation $\sum_{j=1}^{\tau_{all}}$calculates the duration during which the simulation results meet the envelope criteria. $\tau_{all}$ represents the total simulation time. Additionally, *ϵ* represents the permissible error and is set to *ϵ=0.05*.

$\left\{ \begin{aligned} P_{ET}=\frac{\sum_{j=1}^{\tau_{all}} \boldsymbol{1}\left( L_{E_{j}}\leq L_{R_{j}}\leq U_{R_{j}}\leq U_{E_{j}} \right)}{\tau_{all}} \\ U_{E_{j}}=Max\left( f_{i}\left( \tau_{j} \right) \right)\times\left( 1+\epsilon\right),\left\{ j\in\mathbb{Z}^{+} \mid1\leq j\leq\tau_{all} \right\} \\ L_{E_{j}}=Min\left( f_{i}\left( \tau_{j} \right) \right)\times\left( 1-\epsilon\right),\left\{ j\in\mathbb{Z}^{+} \mid1\leq j\leq\tau_{all} \right\} \end{aligned} \right.$ (12)

# Appendix Information B

Night terror is a common childhood parasomnia characterized by abrupt arousal from deep sleep, often sitting upright or screaming, accompanied by confusion, difficulty waking fully, and vivid dreams. In TCM, night terror is attributed to Kidney deficiency; acupuncture at certain points is believed to calm the mind and restore balance. In clinical cases in [40], the selected needling protocol targets KI3 (*Taixi*, *Kidney Meridian*), SP6 (*Sanyinjiao*, *Spleen Meridian*), CV4 (*Guanyuan*, *Conception Vessel*), and BL23 (*Shenshu*, *Bladder Meridian*), applying a mild tonification technique at each point.

In the AMT simulation, parameters are set to reflect both the pathogenesis and treatment strategy for night terror: the Kidney organ state is initialized at 1, while all other organs begin at a healthy value of 3. Stimulation intensities for KI3, SP6, CV4, and BL23 are each set to 1; all non-treated acupoints are assigned a value of 0.


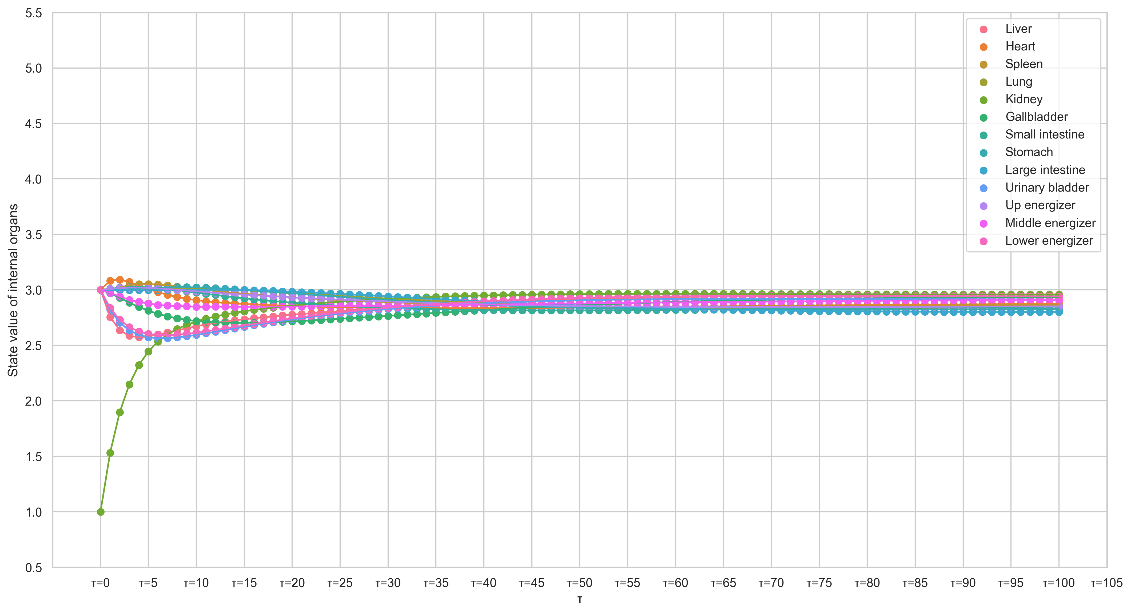


Fig. 6 Dynamic changes in the internal organs during AMT for Night terror.

Using the simulation model developed in this paper, we simulated a clinical case of night terror. The results are illustrated in Fig. 6, demonstrating that acupuncture intervention prompted a rapid recovery of the Kidney state value, which reached the healthy range by $\tau$ = 7. Thereafter, all state values stabilized around 3.0 with no notable fluctuations. This concordance between simulated and clinical outcomes further supports the model’s ability to accurately reproduce AMT’s therapeutic effects in clinical practice.
